# A New Direct Single-Molecule Observation Method for DNA Synthesis Reaction Using Fluorescent Replication Protein A

**DOI:** 10.3390/s140305174

**Published:** 2014-03-12

**Authors:** Shunsuke Takahashi, Shohei Kawasaki, Hidefumi Miyata, Hirofumi Kurita, Takeshi Mizuno, Shun-ichi Matsuura, Akira Mizuno, Masahiko Oshige, Shinji Katsura

**Affiliations:** 1 Department of Chemical and Environmental Engineering, Graduate School of Engineering, Gunma University, Gunma 3768515, Japan; E-Mails: t12801416@gunma-u.ac.jp (S.T.); t09801411@gunma-u.ac.jp (S.K.); b.history12@gmail.com (H.M.); oshige@cee.gunma-u.ac.jp (M.O.); 2 Department of Environmental and Life Sciences, Graduate School of Engineering, Toyohashi University of Technology, Aichi 4418580, Japan; E-Mails: kurita@ens.tut.ac.jp (H.K.); mizuno@ens.tut.ac.jp (A.M.); 3 Cellular Dynamics Laboratory, RIKEN, Saitama 3510198, Japan; E-Mail: tmizuno@riken.jp; 4 Research Center for Compact Chemical System, National Institute of Advanced Industrial Science and Technology (AIST), Miyagi 9838551, Japan; E-Mail: matsuura-shunichi@aist.go.jp

**Keywords:** single-molecule observation, single-stranded DNA, replication protein A (RPA), DNA polymerase, DNA synthesis

## Abstract

Using a single-stranded region tracing system, single-molecule DNA synthesis reactions were directly observed in microflow channels. The direct single-molecule observations of DNA synthesis were labeled with a fusion protein consisting of the ssDNA-binding domain of a 70-kDa subunit of replication protein A and enhanced yellow fluorescent protein (RPA-YFP). Our method was suitable for measurement of DNA synthesis reaction rates with control of the ssλDNA form as stretched ssλDNA (+flow) and random coiled ssλDNA (−flow) via buffer flow. Sequentially captured photographs demonstrated that the synthesized region of an ssλDNA molecule monotonously increased with the reaction time. The DNA synthesis reaction rate of random coiled ssλDNA (−flow) was nearly the same as that measured in a previous ensemble molecule experiment (52 *vs.* 50 bases/s). This suggested that the random coiled form of DNA (−flow) reflected the DNA form in the bulk experiment in the case of DNA synthesis reactions. In addition, the DNA synthesis reaction rate of stretched ssλDNA (+flow) was approximately 75% higher than that of random coiled ssλDNA (−flow) (91 *vs.* 52 bases/s). The DNA synthesis reaction rate of the Klenow fragment (3′-5′exo–) was promoted by DNA stretching with buffer flow.

## 1. Introduction

In general biological research, more than several million molecules have been subjected to conventional analytical methods of biochemistry and molecular biology, such as electrophoretic analysis of the radioisotope-labeled products. These results were the average of a large number of molecules. However, the real behavior of individual biomolecules and the elementary processes of the reactions have not been clarified. In fact, kinetic analysis of the elementary steps of DNA metabolic reactions, such as the binding rate, range of continuous DNA synthesis, and dissociation rate of DNA polymerases, remain insufficiently understood [1]. Direct single-molecule observation reveals individual molecular behavior and DNA-protein interactions with elementary processes of DNA metabolic reactions.

Recently, several studies in single-molecule enzymatic assays have been reported, including: dynamic analysis of the temporal control of lagging-strand synthesis by DNA replication loops [2], single-molecule studies of fork dynamics during *Escherichia coli* DNA replication [3], analysis of molecular brake during DNA replication by DNA primase [4], analysis of base dependence and dynamic disorder by single-molecule kinetics of an exonuclease [5], and real-time single-molecule observation of rolling-circle DNA replication [6]. These studies demonstrated that single-molecule assays were more effective for analyzing DNA–protein interactions in DNA metabolic reactions. Thus, single-molecule assays will provide new insights in the fields of DNA replication, DNA repair, DNA recombination, and RNA transcription [7–13].

To analyze DNA metabolic reactions by direct single-molecule observation, we developed a visualization method for single ssDNA molecules [14]. This method was based on a fusion protein consisting of the ssDNA binding domain of a 70-kDa subunit of replication protein A and enhanced yellow fluorescent protein (RPA-YFP). Using this method, ssλDNA molecules were successfully visualized in microflow channels and easily relabeled by the re-injection of RPA-YFP after stripping RPA-YFP from the complex by the addition of 0.2 M NaCl [15]. These RPA-YFP molecules are thus suitable for real-time microscopic observation of ssDNA regions. This method can be applied to direct observation of DNA synthesis, and the regions labeled with RPA-YFP may be quenched because of the release of RPA-YFP that accompanies the progression of DNA synthesis. However, it is difficult to distinguish between quenching due to the release of RPA-YFP by a DNA synthesis reaction and that due to the breakdown of template DNA by exonuclease activities of DNA polymerases.

In this study, we report a new direct single-molecule observation method for DNA synthesis reactions, and applied to the Klenow fragment (3′-5′ exo–) which is a mutant form (D355A, E357A) of DNA polymerase I. Because the Klenow fragment (3′-5′ exo–) lacks both 5′-3′ and 3′-5′ exonuclease activities [16–19], quenching due to the breakdown of template DNA by exonuclease activities of DNA polymerase is negligible. In addition, the new developed method successfully determined the DNA synthesis reaction rate of the Klenow fragment (3′-5′ exo–) through the direct observation of single-stranded region labeled with RPA-YFP of both hydraulically stretched template DNA (+flow) and random coiled template DNA (−flow) via buffer flow. The effects of DNA tension on DNA synthesis reactions are discussed in this study.

## 2. Experimental Section

### 2.1. Proteins and Chemical Reagents

RPA-YFP was prepared using a pET32-eYFP vector and Rosetta (DE3) as described previously [14]. The Klenow fragment (3′-5′ exo–) was purchased from New England Biolabs (Ipswich, MA, USA). Water was purified using a Millipore Milli-Q water system (Billerica, MA, USA). Other reagents used in this study were of analytical grade and were obtained from Wako (Osaka, Japan) or Sigma Aldrich (St. Louis, MO, USA).

### 2.2. Preparation of ssλDNA and Oligonucleotide-Annealed ssλDNA

To prepare thiol-modified ssλDNA molecules, ssλDNA molecules were annealed using a 25-mer oligonucleotide at the 3′ terminal end. This oligonucleotide sequence was 5′-CGT AAC CTG TCG GAT CAC CGG AAA G-3′ (Japan Bio Services; Saitama, Japan). The reaction mixture consisted of an annealing buffer [20 mM Tris-HCl (pH 8.0), 50 mM KCl, 1.5 mM MgCl_2_, 1 mM EDTA (pH 8.0)], 4 fmol of thiol-modified λDNA, and 100 pmol of 25-mer oligonucleotide. The reaction mixture was incubated according to the following heat denaturation program: 94 °C for 6 min, 58 °C for 1 min, and cooling to 4 °C. Detailed information of the preparation of thiol-modified λDNA is provided in our previous paper [15].

### 2.3. Optical set-up and Temperature Control

DNA molecules were observed using a fluorescence microscope (ECLIPUSE TE-2000U; Nikon, Tokyo, Japan) equipped with a 100×, 1.3 numerical aperture (NA) oil immersion objective lens (PlanApo; Nikon). The excitation light intensity was controlled by neutral density (ND) filters. The excitation light and emission light were selected using a filter set B-2A (blue excitation light, EX450-490, DM505, and EM520) or a filter set G-2A (green excitation light, EX510-560, DM575, and EM590) purchased from Nikon. Fluorescent images of single-stranded and double-stranded DNA molecules stained with YFP (excitation 513 nm, emission 527 nm) and SYTOX Orange (excitation 547 nm, emission 570 nm) were visualized using a high sensitivity Watec Monochrome CCD camera (WAT-120N+; Watec, Yamagata, Japan) and recorded using mAgic TV5 (I-O DATA; Ishikawa, Japan). The length of individual DNA molecules was also determined using imageJ. The temperature of the DNA synthesis reaction was controlled by a heat plate (MATS-505RA20; Tokai Hit, Shizuoka, Japan).

### 2.4. Single-Molecule DNA Synthesis Reaction by DNA Polymerase

**Figure 1.**
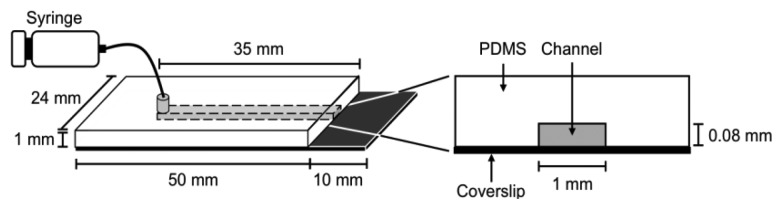
Overview of the microflow channel used in this study.

**Figure 2.**
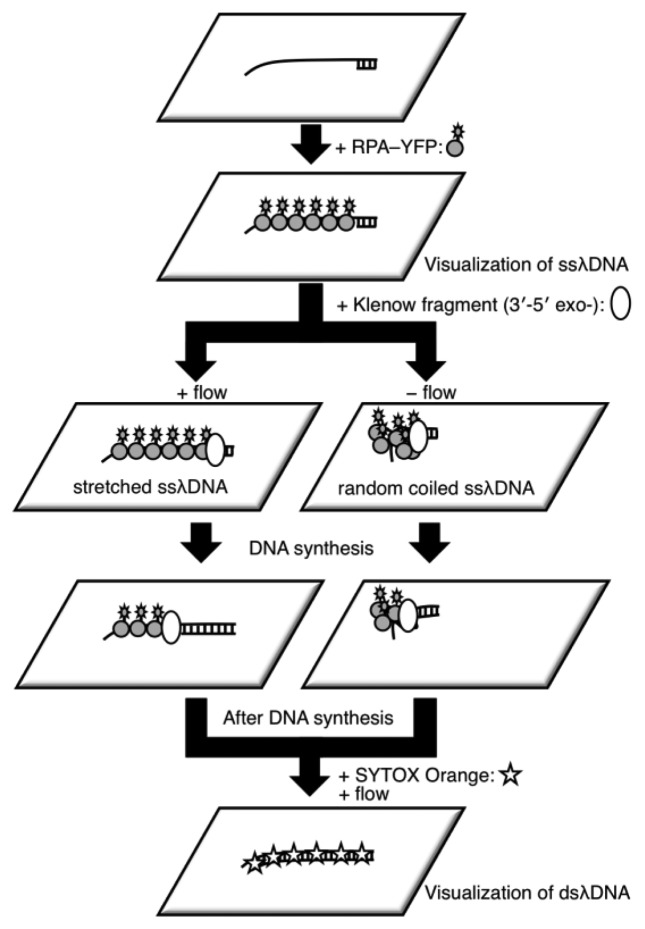
Schematic illustration of the single DNA synthesis reaction. One end of ssλDNA was immobilized on a glass surface and then injected into RPA-YFP molecules bound to the ssλDNA molecules. For stretched ssλDNA, single-molecule observation was performed with reaction buffer flow. In contrast, for random coiled ssλDNA, the reactions were performed without buffer flow. When a fluorescent image of ssλDNA was captured, buffer flow was transiently applied for a few seconds. Following image capture, the flow was stopped, and the immobilized ssλDNA acquired a random coiled form. After completion of the DNA synthesis reaction, SYTOX Orange was injected to stain double-stranded regions of λDNA, which were then visualized. Fluorescent images for both ssλDNA and dsλDNA were captured in the same microscopic field.

It is difficult to measure the contour length of DNA molecules in a random coiled state. Thus, microflow channels were used to stretch DNA molecules as shown in Figure 1. A microflow channel inlet was connected to a syringe (1725TLL; Hamilton, Reno, NV, USA) containing a common buffer [50 mM Tris-HCl (pH 7.8), 10% glycerol, 0.1% Tween 20, 2.5% 2-mercaptoethanol]. The syringe was installed on a syringe pump (KDS-100; KD Scientific Inc., Holliston, MA, USA). In the following experiments, the flow rate was adjusted to 25 μL/h unless otherwise stated. The experimental procedure of a single-molecule DNA synthesis reaction by DNA polymerase is shown in Figure 2.

In the experimental procedure for analyzing the DNA synthesis reaction by the Klenow fragment (3′-5′exo–), thiol-modified ssλDNA molecules were first injected into the microflow channel for 30 min. The glass surface was pretreated with dichlorodimethylsilane for one-end immobilization of individual thiol-modified ssλDNA molecules [13,15,20]. The ends of the thiol-modified ssλDNA molecules were immobilized on the glass surface during this period. The common buffer containing 1% bovine serum albumin (BSA) was then injected into the microflow channel for 30 min both to block the glass substrate and remove free ssλDNA molecules. A common buffer (200 μL) containing 0.5 nmol RPA-YFP was injected into the microflow channel for 30 min. During this period, RPA-YFP molecules bound to ssλDNA molecules. At this time, the heat plate was set at 37 °C during this process. The common buffer was then injected into the microflow channel to remove excess RPA-YFP so that signals from the free fluorescent molecules did not interfere with fluorescent observation of the single-stranded region of the template DNA.

Next, a DNA synthesis reaction buffer [50 mM NaCl, 10 mM Tris-HCl (pH 7.9), 10 mM MgCl_2_, 1 mM dithiothreitol] containing 0.3 mM of each dNTP, 10% glycerol, 0.1% Tween 20, and 12.5 U of the Klenow fragment (3′-5′ exo–) was injected into the microflow channel until the entire chamber was uniformly filled with the buffer. Direct observation of the DNA synthesis reaction was preformed for both stretched ssλDNA and random coiled ssλDNA. The stretched ssλDNA molecules were observed under a flow rate of 25 μL/h. Fluorescent images were captured every 1 to 2 min. For random coiled ssλDNA, the buffer flow was transiently applied for a few seconds at a flow rate of 25 μL/h only when the fluorescent images of ssλDNA were captured. After completion of the DNA synthesis reaction, 0.3 μM SYTOX Orange (intercalation type fluorescent dye; Life Technologies, Grand Island, NY, USA) was injected into the microflow channel for 30 min to stain the double-stranded regions of λDNA, which were then visualized. Fluorescent images for both ssλDNA and dsλDNA were captured in the same microscopic field.

## 3. Results and Discussion

**Figure 3.**
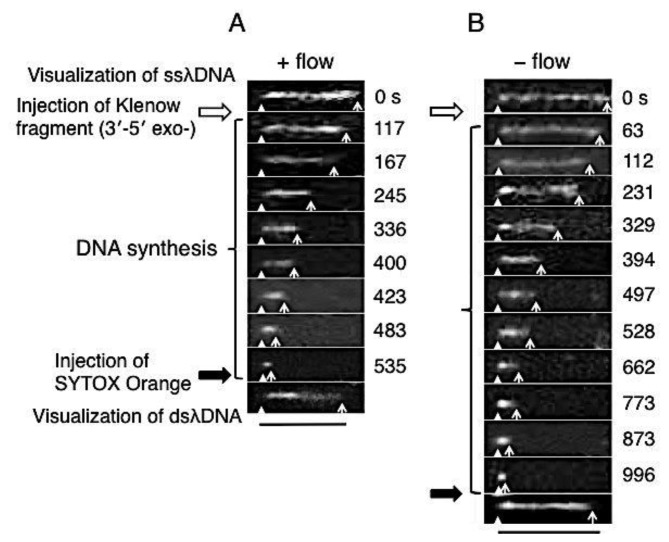
Sequential photographs of the direct temporal observation of the DNA synthesis reaction by the Klenow fragment (3′-5′ exo–). In presence of Klenow fragment (3′-5′ exo–) with buffer flow (**A**), in presence of Klenow fragment (3′-5′ exo–) without buffer flow (**B**). White triangles and white arrows indicate the positions of the immobilized and free ends of stretched ssλDNAs, respectively. Scale bar = 10 μm.

Direct observation results of the DNA synthesis reaction by the Klenow fragment (3′-5′exo–) are shown in Figure 3. In these sequential photographs, single-stranded regions of λDNA were visualized by RPA-YFP molecules under stretched (+flow) and random coil (−flow) conditions, as shown in Figures 3A and B, respectively. Fluorescent images were captured every 1 to 2 min during the DNA synthesis reaction. The observed ssλDNA molecules were stretched by continuous buffer flow (Figure 3A) or by transient buffer flow supplied only during the capture of the DNA synthesis reaction (Figure 3B).

For one-end immobilized ssλDNA molecules, the white triangles and white arrows denote the positions of the immobilized end and free end, respectively. The single-stranded regions of the template λDNA molecule monotonously shortened during the DNA synthesis reaction. The DNA synthesis reaction was completed in 535 s under the stretched condition (+flow) (Figure 3A). On the other hand, the DNA synthesis reaction was completed in 996 s under the random coiled condition (−flow) (Figure 3B).

**Figure 4.**
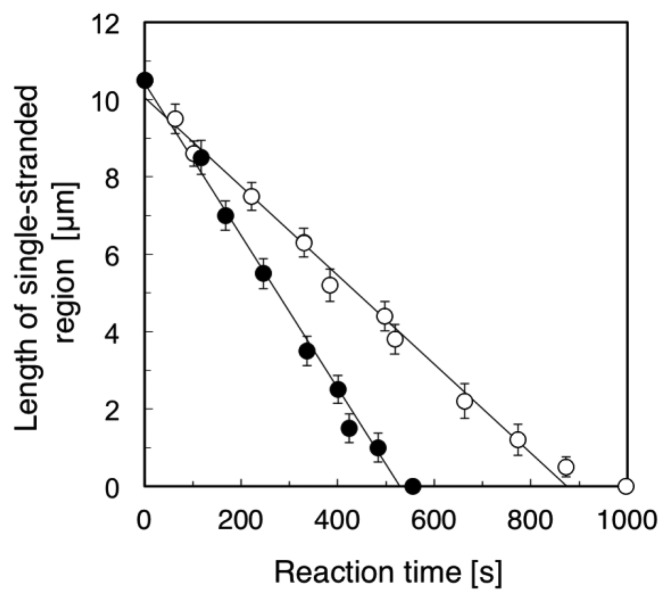
Time course of length of the single-stranded region on stretched ssλDNA and random coiled ssλDNA during the DNA synthesis reaction by the Klenow fragment (3′-5′ exo–). Closed circle (●) and open circle (○) denote the length of the single-stranded region on stretched ssλDNA and that on random coiled ssλDNA, respectively. The error bars represent the standard deviations in the length of the single-stranded region of five different ssλDNA molecules through direct observation of DNA synthesis reaction under the stretched and the random coiled conditions, respectively.

Figure 4 summarizes the time course of the length of the single-stranded region (the length of the RPA-YFP labeled region). We examined five different ssλDNA molecules for direct observation of the DNA synthesis reaction under the stretched (+flow) and random coil conditions (−flow), respectively. Based on the results, we calculated the standard deviations in the length of the single-stranded region, and attached the error bars in Figure 4. The DNA synthesis reaction rate of stretched ssλDNA (+flow) was 1.18 μm/min (closed circles in Figure 4). On the other hand, the DNA synthesis reaction rate of random coiled ssλDNA (−flow) was 0.68 μm/min (open circles in Figure 4). By measuring the length of DNA under stretched conditions, the lengths of constantly stretched ssλDNA molecules and transiently stretched ssλDNA molecules (random coiled structure) were determined to be 10.5 μm. These observed lengths were used to derive a coefficient to convert the stretched length of ssλDNA into the number of bases, which was 4,619 bases/μm for ssλDNA in both states (ssλDNA: 48,502 bases).

Based on the number of bases/μm, the DNA synthesis reaction rate of the stretched ssλDNA (+flow) was estimated to be 91 bases/s. On the other hand, the DNA synthesis reaction rate of random coiled ssλDNA (−flow) was estimated to be 52 bases/s. It was reported that the DNA synthesis reaction rate of DNA polymerase I (Klenow fragment) was 50 bases/s in the assay of ensemble molecules [21]. The DNA synthesis reaction rate of random coiled ssλDNA (−flow) was nearly the same as that measured in the ensemble molecule experiment (52 *vs.* 50 bases/s). This result suggested that the random coiled form of DNA (−flow) reflected the DNA form in the bulk experiment in the case of DNA synthesis reactions. Therefore, our single-molecule experiments were consistent with the DNA synthesis assay of ensemble molecules.

We also compared the DNA synthesis reaction rates of stretched ssλDNA (+flow) and random coiled ssλDNA (−flow) at the single-molecule level. The DNA synthesis reaction rate of stretched ssλDNA (+flow) was approximately 75% higher than that of random coiled ssλDNA (−flow) (91 *vs.* 52 bases/s). This suggested that the DNA synthesis reaction rate was significantly promoted by higher DNA tension. The effects of tension on DNA metabolic reaction have been previously studied, e.g., the effects of template tension on DNA polymerase [7,8], RNA polymerase [9], and exonuclease activity [7,13] and DNA tension dependence on restriction enzyme activity [10,11]. In the case of exonuclease III activity [13], the digestion rate of stretched DNA was higher than that of relaxed DNA. The DNA synthesis rate of T7 DNA polymerase with stretched template DNA until approximately 6 pN was twice as high as that with the relaxed form of DNA without tension [7].

The previously published single-molecule method described by van Oijen's group [6], which involves probing the elongation of many DNA strands simultaneously, is similar to our single-molecule method. In their method, the product length of synthesized DNA on rolling-circle DNA replication was monitored with intercalating stain. On the other hand, our single-molecule method was directly observed by the template ssλDNA labeled with RPA-YFP on the DNA synthesis reactions under the stretched (+flow) and the random coil (–flow) conditions. Thus, we suggest that our single-molecule method can be applied to an advantage as direct observation of various DNA synthesis reactions including DNA metabolic reaction. With regard to enzyme activity with/without buffer flow, Yao *et al.* demonstrated that the product length of synthesized DNA on single-molecule rolling circle replication by *E. coli* polymerase III replicase with buffer flow was approximately 62% longer than that without buffer flow [12]. Our results also demonstrated that the DNA synthesis reaction rate of the Klenow fragment (3′-5′ exo–) was promoted by the hydraulic stretching of template single-stranded DNA with buffer flow.

## 4. Conclusions

In this study, we develop a new method to observe single-molecule DNA synthesis reactions in microflow channels, and applied to DNA synthesis by the Klenow fragment (3′-5′ exo–). The DNA synthesis reaction rates both for stretched ssλDNA (+flow) and random coiled ssλDNA (−flow) were successfully determined as 91 bases/s and 52 bases/s, respectively. The DNA synthesis reaction rate of random coiled ssλDNA (−flow) was almost the same as that measured in the ensemble molecule experiment (52 *vs.* 50 bases/s). This result indicates that our single-molecule experiments were consistent with the assay of ensemble molecules, and our method can be applied to effects of DNA form on DNA synthesis reaction.
